# The Effects of Ayahuasca on Psychological Disorders: A Systematic Literature Review

**DOI:** 10.7759/cureus.55574

**Published:** 2024-03-05

**Authors:** Reena Sheth, Esha Parikh, Kunmilayo Olayeye, Kylie Pfeifer, Deepesh Khanna

**Affiliations:** 1 Foundational Sciences, Nova Southeastern University Dr. Kiran C. Patel College of Osteopathic Medicine, Fort Lauderdale, USA; 2 Foundational Sciences, Nova Southeastern University Dr. Kiran C. Patel College of Osteopathic Medicine, Clearwater, USA

**Keywords:** mystical experience, personality changes, ritual, childhood trauma, ptsd, eating disorders, substance abuse, mood and anxiety, depression prevention, ayahuasca

## Abstract

Ayahuasca is an original Amazonian brew made from the vines and leaves of* Psychotroa viridis *and* Banisteriopsis caapi. *Both *P. viridis and B. caapi* give this brew its unique psychedelic properties which have been revered over centuries. In recent years, ayahuasca has gained attention as a potential therapeutic tool for mental health disorders, including substance abuse and depression. The uniqueness of ayahuasca’s therapeutic potential is that it is an amalgamation of its biochemical makeup and the ritual guided by a shaman, along with the interpretation of the participant of their experience. The boom of “ayahuasca tourism” has brought forth testimonies of feeling “cured” of depression, and substance abuse and an improvement in overall well-being. This systematic literature review focuses on summarizing the recently available research on the effectiveness of ayahuasca as a treatment for depression, anxiety, substance abuse, eating disorders, and post-traumatic stress disorder. It also focuses on understanding the effects it has on personality traits that play a significant role in the manifestation of the above-listed mental health conditions effects. Additionally, the review investigates the importance and role the ritual itself plays, often described as the “mystical experience”. This systematic literature review aims to explore the current state of knowledge regarding the use of ayahuasca for numerous mental health conditions by analyzing medical research papers published no earlier than September 2017 to no later than May 2023 from Google Scholar and PubMed. A total of 43 articles met the criteria and were used for detailed analysis. This review will synthesize the findings of the studies, examining the potential therapeutic effects of ayahuasca on multiple mental health disorders, the significance of the “mystical experience,” and the mechanisms of action underlying its effects. Through the review, ayahuasca proves to be a worthwhile therapeutic tool that if used in the right setting influences mind, body, and spirit. It is important to note that most studies used in this article relied on surveys and self-reporting proving to be a limitation as no clear standard has been achieved to test the efficacy of ayahuasca. The respect for the culture and origin needs to be retained as Western medicine dwells deeper into ayahuasca’s benefits.

## Introduction and background

As of 2022 WHO officially released a warning statement indicating a 25% increase in prevalence of anxiety and depression worldwide. The increase in negative emotions has been associated with rapidly accelerating substance abuse and poor eating and sleeping habits [1]. This crisis has further been strained by the limited access to care. In 2020 approximately less than one mental health professional for every 10,000 people was estimated [2]. The increased burden on both patients and healthcare calls for an urgent need for holistic treatment that can help resolve this multidisciplinary crisis. 

Hallucinogens are a group of psychoactive substances that have been known to elicit altered perception, mood, and cognition. Throughout history, hallucinogens have been constantly used across cultures for both medicinal and religious practices [3]. While they are classified as Schedule 1 substances in the United States, there was a rapid increase in hallucinogen use of about 8% in 2021 [4]. This has been the highest reported usage since the discovery of the hallucinogenic properties of ayahuasca in 1988. Similar trends were seen in the UK with a shift towards micro-dosing to improve well-being [5].

Ayahuasca, an Amazonian brew made from vine and leaves of *Psychotroa viridis* and *Banisteriopsis caapi* has specifically gained increased popularity. The brew is traditionally prepared by placing the washed stem scraps of *B. caapi* and leaves of *P. viridis* in a large cauldron and pounded with a wooden mallet. *B. caapi* contains harmala alkaloids and β-carbolines, and *P. viridis* contains N, N dimethyltryptamine (DMT), a potent psychedelic [6]. The β-carbolines are reversible inhibitors of A-type isoenzymes of monoamine oxidase (MAO) and selective serotonin reuptake inhibitor effects [6,7]. *P. viridis* on the other hand is a source of DMT that is a serotonin receptor agonist. The inhibitor effects on monoamine oxidase prevent DMT from being broken down, hence allowing it to be orally active [6,7]. 

This chemical makeup of ayahuasca has been shown to have significant antidepressant, anti-addictive, and anti-anxiety effects along with an overall improvement in well-being and mindfulness. Additionally, ayahuasca has demonstrated an overall increase in quality of life amongst users suggesting it is a beneficial therapeutic agent for post-traumatic stress disorder (PTSD) and eating disorders [8].

This Amazonian brew has been said to have been used by multiple Peruvian tribes for over millennia. The importance of using the ayahuasca vine has not been the chemical makeup but rather that it adds wisdom to the journey one takes participating in the ceremony. With the increasing interest in the Western population, one would expect a significant rise in the integration of ayahuasca into Western practices. However, most users do not differentiate it from the traditional ritual itself. They do not look at ayahuasca as a drug but rather as a path to healing, to connect with something beyond. Ingestion of ayahuasca is always guided by a curandero (spiritual leader) in a group setting. The curandero first understands the participants, and their expectations and starts the journey to treat their spirit. The curandero starts the ceremony at night by calling on each participant to drink the tea. In complete darkness, the healer begins to sing a healing song called “icaros.” Through music, the healer communicates with the spirits and asks for their help through this healing journey. Simultaneously participants continue to navigate their journey. The curandero does not limit themselves to music, instead use a variety of tools including breath, hands, tobacco, and aqua to individualize the journey to each participant’s need. At the end of the ceremony the healer protects the spirits, lights are turned on and participants can leave. This ceremony alone does not make up for the use of ayahuasca. The entire ceremony includes diet, purgatives, vapor baths, plant baths, and faith. Participants usually have visions and altered states of mind. The effect usually lasts about four to six hours. Some participants report nausea and vomiting; however, this is described as the healing of the body, a type of cleansing of your physical self. Whereas the feeling of anxiety, confronting hallucinations, and fear is described as healing of the mind, participants are taken back in time to the root cause and stand up to it [9].

WHO has reported around 280 million people suffering from depression with about 700,000 people dying from suicide in 2023 [10]. In addition, one-third of these patients do not respond to currently accepted depression treatment [10]. A single dose of ayahuasca has shown significant therapeutic potential in patients who are resistant to treatment [11]. However, the current legal restriction has limited the ability to assess the efficacy of ayahuasca as a pharmaceutical option for depression which can be clinically prescribed. Not only are there legal hurdles, but also social stigma. It seems that some studies use a dosing method measured in metric units, while others use a dosing method based on administration. Moreover, most information is coming from countries that are more open to psychedelic usage like Brazil. The present study aims to understand if ayahuasca has an effect in more than one way and to tie these different avenues that multiple researchers have discovered into a comprehensive understanding of how ayahuasca can be a possible treatment for depression and other psychological disorders defined in the *Diagnostic and Statistical Manual of Mental Disorders, Fifth Edition, Text Revision *(DSM-V-TR). 

Substance abuse and addiction disorders are an epidemic that is closely associated with depression and anxiety. Current treatments include group therapy, nicotine replacement therapy (bupropion and varenicline), methadone, buprenorphine, naltrexone, acamprosate, and disulfiram. While these are routinely used the adverse effects and adherence to treatment create limits. This study aims to answer how ayahuasca as a psychedelic therapy compares to these traditional therapies and understand the multifactorial effect that makes it a beneficial therapy and its efficacy for long-term cessation. 

With the destruction of stigmas surrounding psychedelics, ayahuasca-born tourism has rapidly increased in recent years [12]. These centers are no longer exclusive to Peru but are seen increasingly across South America. The mainstreaming of the experience by celebrities like Will Smith describing it as the “unparalleled greatest feeling,” or Mike Tyson advocating for increased accessibility to ayahuasca after it “cured” him. Tyson emphasizes that his wealth and status have allowed him to personally explore the medicinal effects of ayahuasca, an experience that has helped numerous wealthy groups improve vis a vis cure their mental health, while much of the lower class struggles to afford their necessities. Countries like Australia have already begun working on projects like the "Global Ayahuasca Project" and plan to commence clinical trials shortly on ayahuasca, evaluating its effectiveness for alcohol dependence. The increasing support and research for ayahuasca, coupled with the rise of the mental health crisis in the United States, warrants a genuine look toward ayahuasca as a viable treatment option [13,14].

The primary aim of this systematic review is to provide an updated overview of facts and any hypotheses of ayahuasca’s therapeutic potential along with its mechanism of action. Mental illnesses focused on this paper are depression, substance abuse disorder, post-traumatic stress disorder, eating disorders, and anxiety. The review will also focus on the effects of ayahuasca on the well-being of participants including an increase in cognitive creativity, mindfulness, and simultaneously ego death. In addition, the review will discuss the importance of the influence the ritual itself has on the therapeutic potency of ayahuasca.

## Review

Methodology

**Figure 1:**
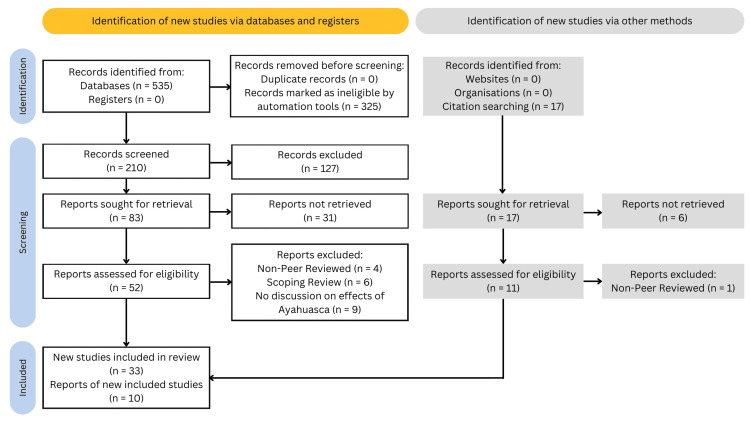
Study selection PRISMA flow diagram [15] PRISMA: Preferred Reporting Items for Systematic Reviews and Meta-Analyses

This systematic literature review focuses on the current evidence on the therapeutic effects of ayahuasca on mental health conditions, including depression, anxiety, substance abuse, eating disorders, PTSD, personality changes, and the impact of the ayahuasca ritual itself. The databases used were Google Scholar, PubMed, and EBSCO host. This review was conducted under Preferred Reporting Items for Systematic Reviews and Meta-Analyses (PRISMA) Guidelines. The search terms: “Ayahuasca,” “Depression,” “Substance Abuse,” “Eating Disorders,” “PTSD,” “Mechanism of Action,” “Anxiety,” “Ritual,” “Ceremony,” “Personality Changes,” “Mystical Experience,” “Settings,” and “Cessation,” were used to find relevant articles. Inclusion criteria for this study permitted original studies conducted on human participants, published between September 2017 and May 2023, in any part of the world, cross-sectional studies with a sample size greater than 30, experimental studies, longitudinal studies, case studies reporting on the effects of ayahuasca on mental health conditions, and studies reporting on the mechanisms of action of ayahuasca on mental health conditions, including neurobiological and psychological mechanisms. The aforementioned researchers carried out all searches. Studies were excluded if they focused only on a specific subtopic of a mental disorder, published in non-peer-reviewed books and documents, or classified as meta-analyses or scoping reviews. The systematic review of pertinent studies and the extraction of important data, such as study design, sample size, participant demographics, intervention details, outcome measures, and findings, were followed under PRISMA criteria. The scope of findings due to the variety of studies and the exclusion of meta-analyses or scoping reviews are limitations of this methodology. A total of 43 studies were included in this review. Data was synthesized to identify patterns and themes across eligible studies with the process of selection illustrated in Figure 1.

Discussion

This discussion compiles the recent research done on ayahuasca and its role in psychological disorders as a treatment method. Detailing the various biochemical, medicinal, and spiritual uses of ayahuasca as seen in medical research around the world. In this discussion, we have explored ayahuasca as a treatment for depression, anxiety, substance abuse disorder, eating disorders, PTSDs, and overall mental wellness while also exploring the holistic aspect of ayahuasca in the context of a spiritual ritual and the biochemical mechanism of action. Additionally, this discussion considers the concept of the “mystical experience” which in recent research has proven as an essential ideology in truly understanding the power behind ayahuasca as a treatment for common psychological disorders.

Mechanism of Action of Ayahuasca

Ayahuasca is a traditional psychoactive brew composed of the leaves of the *P. viridis* bush and scraps of the stem of the *B. caapi* vine, which is indigenous to the Amazonian Basin. The method of making this brew is not entirely exact with over 100 additive plants that have been incorporated in its preparation over time, however, the two main ingredients stay the same. *B. caapi* provides several β-carboline alkaloids, such as harmine, tetrahydroharmine (TTH), and harmaline. *P. viridis* supplies the brew with the main psychoactive ingredient DMT. Which is a serotonin receptor agonist normally metabolized by peripheral monoamine oxidase A (MAO-A). The B-carboline alkaloids temporarily inhibit MAO-A, allowing DMT to remain active in the body longer and access the central nervous system. TTH in addition to being an MAO-A inhibitor, blocks serotonin reuptake, amplifying the effects of DMT. TTH readily crosses the blood-brain barrier due to its hydrophobic makeup and low molecular weight [16]. This subtopic aims to briefly explore the biochemical and neurological possibilities associated with this ancient mystical brew.

Biochemical targets: The active ingredients of ayahuasca have a variety of effects on the body. Ayahuasca’s pharmacological profile is extremely complex and is not entirely understood. There is a diverse range of systems of which ayahuasca’s common active compounds DMT, TTH, harmine, and harmaline affect. DMT activates the serotonergic systems within the body as an agonist for 5- HT 1A, 2A, and 2C receptors increasing anti-depressant effects, stress adaptation, and neuroprotection [17,18]. The MAO-A inhibition and blockage of serotonin reuptake via B-carbolines increase the serotonin levels which increase brain-derived neurotrophic factor (BDNF) and VEGF concentrations, hypothalamic-pituitary-adrenal axis activity (HPA) and antidepressant effects [17,18]. The dopaminergic effects are due to MAO-A inhibition, which augments D1 and D2 activity, which increases motivation and decreases anhedonia. The glutaminergic effects are directly related to 5-HT 1A activation of AMPA receptors in the brain which increases BDNF concentrations. DMT serves as a sigma-1 receptor agonist, which increases cell survival and proliferation, along with having neuroprotective benefits [17]. It has been hypothesized that the importance of sigma-1 receptors is attributed greatly to ayahuasca's effects on memory retrieval and modulating fear. Sigma-1 receptors have been hypothesized to be associated with the repression of memories, and since the MAO-A inhibitors in the brew target, these receptors suggest that ayahuasca may have anti-amnesic properties [18,19]. Lastly, the neuroendocrine effects of ayahuasca normalize HPA axis activity which increases stress adaptation and regulates cortisol levels [20]. Ayahuasca also increases growth hormone and prolactin in the body [20]. In one randomized control trial ayahuasca reduced levels of C-reactive protein (CRP) in both depressive and healthy patients, proving that ayahuasca has an anti-inflammatory component as well [21]. The vastly impressive biochemical profile of ayahuasca suggests that it is still a pharmaceutical enigma, however, the challenge of understanding and harnessing the biochemical facets of this psychoactive brew could produce a medical novelty. The synergistic effects of DMT, TTH, harmaline, and harmine are what make this ancient brew so diverse and worth studying. 

The effects of DMT on the brain: The study of the main psychoactive compound in ayahuasca, DMT, has been proven to have profound neurological effects through multiple electroencephalography (EEG) studies in the past five years. The concept of mystical experience brought up through ayahuasca use could be possibly explained by the effect of DMT on brain waves, as the aforementioned biochemical targets have failed to provide a definitive explanation for this phenomenon. One study focused on the effects of DMT in naturalistic settings and found that there is a statistically significant decrease in alpha waves throughout all scalp locations with a simultaneous increase in delta waves. Additionally, an increase in gamma power was noted, suggesting that this wave may contribute to the mystical experience of ayahuasca. The study associated certain EEG readings with specific questionnaires that participants had to fill out regarding their experience. The researchers noted that beta oscillations correlated with “cognition” while gamma oscillations correlated with “experience of unity,” “disembodiment,” “complex visual imagery,” and “transcendence of time and space.” While the most significant channels were noted in the occipital and central regions [22]. The findings of this study are corroborated by another EEG study done on participants who were dosed with intravenous DMT. The findings of decreased alpha wave oscillatory power were the most significant in this study with a mention of decreased beta bands and an increase in spontaneous signal diversity. This study also supports an increase in delta waves and saw an increase in theta waves as well in their subjects. This study attributes the transcendent feeling, a characteristic of the ayahuasca use, to the reduction of alpha waves [23]. In a study focusing on the EEG activity of participants who ingested ayahuasca, similar findings were seen. Synchronization of gamma bands in the parietal, occipital, and frontal cortices were observed for facial recognition tasks and suggested that this band is imperative for understanding the visual hallucinations associated with ayahuasca use. This study also suggests that the typical organization of the brain is disrupted allowing for strong and far-reaching functional connections to emerge which are not present in the natural state of mind [24]. Thus, the potential effects of ayahuasca are due to a synergistic effect on both the biochemical pathways and neurological pathways of the human brain, suggesting a promising wide range of therapeutic potential for the major psychological disorders that plague our modern society.

Depression

In all studies included in this subtopic ayahuasca has measurable antidepressant properties with each study discussed summarized in Table 1. The potential use of ayahuasca specifically for moderate-to-severe depression will be divided into subtopics of its effect on suicidal ideation, potential biochemical mechanisms, and the importance of mystical experiences [8]. In more than one study it is mentioned that the combination of both biochemical mechanisms and the unquantifiable “mystical experience” contributes to the lasting anti-depressant effects of ayahuasca [25]. It is proposed that a single dose of ayahuasca could have lasting effects of almost a year, with the most rapid decrease in symptoms seen right after the ayahuasca dose and the lowest scores reported almost seven days after the dose [11]. A cross-sectional study of 11,912 participants suggests that even across cultures around the world, ayahuasca use is associated with antidepressant effects even after one dose. As one of the only studies with such a large group of participants ranging from one use to consistent ayahuasca use it helps suggest that the use of ayahuasca is strongly associated with antidepressant effects, particularly in a safe and explorative setting. This study suggests lower rates of depression and anxiety with longer lifetime use of ayahuasca [25]. In the one clinical trial on ayahuasca to date, depression symptoms reduced for each participant who was dosed with ayahuasca, although individual symptom reduction varied [11]. Other studies focusing on suicide, which is strongly correlated with depression, saw a large statistical decrease in non-suicidal depressive symptoms associated with ayahuasca. This study also highlighted the rapid action of ayahuasca, which is not seen with any other available conventional antidepressant therapy, which could benefit those with suicidal ideation quickly by decreasing emotional dysregulation [26]. In a longitudinal study conducted over a year after one ayahuasca dose, depression symptoms decreased and helped those who previously had treatment-resistant depression be able to lessen their depression through antidepressants, other psychedelics, and therapy. This raises the possibility that ayahuasca may inspire depression patients to seek out alternative treatments or re-try previous failed treatments due to a new outlook on their disorder [8]. The effects seen with just one dose of ayahuasca within one setting prove promising compared to conventional therapies which require multiple or constant doses with adverse effects. The multiple combined effects of increased cortisol levels, brain-derived neurotrophic factor (BDNF), anti-inflammatory effects, and stimulation of the sigma 1 receptor along with positive mystical experiences during ayahuasca use and the after-effects all contribute to ayahuasca’s potential as an antidepressant for those with significant depression with poor remission by current pharmaceuticals available [26]. The details of the above-mentioned studies are summarized in Table 1.

**Table 1:** Methods and administration of studies for anti-depressant effects

Study	# of doses	Method of administration	Length of study	Depression scale used	Number of people	Result
Van Oorsouw K et al. (2022) [8]	1	Mimosa hostilis + Peganum harmala + Banisteriopsis muricata; caapi + Peganum harmala + Mimosa hostilis	1 year	Beck’s Depression Inventory (BDI)	20 participants with clinical depression (>13 on BDI)	BDI depression scores significantly reduced over time, with 71% in remission
Sarris J et al. (2021) [25]	Variable, self-reported	Variable, self-reported	2017-2020 (Global Ayahuasca Project)	Kessler-10 (K-10) Psychological Distress Scale. Participants were asked if they had been diagnosed by a health professional with depression or anxiety. Patient Health Questionnaire for Depression and Anxiety	11,912; 1,580 with depression	94% believed that their depression had improved either ‘a bit’ (15%), ‘very much’ (46%), or was ‘completely resolved’ (32%) due to their consumption of the brew
Palhano-Fontes F et al. (2019) [11]	1	Single ayahuasca batch prepared and provided free of charge by a branch of the Barquinha church based in Brazil. Harmine + Harmaline + Tetrahydroharmine	1 week	Montgomery-Asberg Depression Rating Scale (MADRS) and the Hamilton Depression Rating Scale (HAM-D)	29	Evidence of rapid antidepressant effect after a single dosing session with ayahuasca when compared with placebo
Zeifman RJ et al. (2019) [26]	1	Harmine + Harmaline + Tetrahydroharmine	1 week	Referral from outpatient psychiatric units and advertisement. Interested participants received a full clinical assessment by a psychiatrist in order to determine eligibility	29	No statistical decrease in suicidal ideation in the treatment group. Decreases in non-suicide-related depressive symptoms were greater than those found for suicidality
Rossi GN et al. (2022) [17]	1	A single batch of ayahuasca was prepared by a branch of the Barquinha church. Harmine + Harmaline + Tetrahydroharmine	2 days	MADRS & HAM-D	Treatment-resistant depression patients (n = 28) and healthy controls (n = 45)	Reductions of C-reactive protein (CRP) and lower depressive symptoms at 48 hours after substance ingestion. No correlation of IL-6 with decreased depressive symptoms. Increased brain-derived neurotrophic factor (BDNF) correlated with less depressive symptoms
De Almeida RN et al. (2019) [18]	1	A single batch of ayahuasca was prepared by a branch of the Barquinha church. Harmine + Harmaline + Tetrahydroharmine	2 days	Selected patients were in a current moderate to severe depressive episode at screening by the Hamilton Depression Rating Scale (HAM-D≥17) and MADRS	71 participants (Healthy control = 43, and muscular dystrophy patients = 28)	Basal hypocortisolemia and in patients with treatment-resistant depression, compared to healthy controls. Depressive patients who took ayahuasca had higher levels of cortisol (normal in healthy control) than placebo

Biochemical effects: Several observed mechanisms correlate with ayahuasca and may contribute to the anti-depressive effect seen with its use. One study analyzes the anti-inflammatory effects of ayahuasca, specifically CRP and interleukin-6 (IL-6), and their correlation with BDNF and cortisol levels in the body. It was shown that in both treatment-resistant depression and healthy control participants ayahuasca significantly decreased the amount of CRP, and that pretreatment treatment-resistant depression participants started with elevated CRP in their bloodwork. The decrease in CRP correlated with a decrease in the Montgomery-Åsberg Depression Rating Scale (MADRS) scores among those with depression. Treatment-resistant depression has been associated with mild chronic systemic inflammation. In this study, a single dose of ayahuasca, and not the placebo, reduced both depressive symptoms and systemic inflammation of treatment-resistant depressive patients two days after the treatment [21]. Thus, possibly shedding light on the biological mechanisms that underlie the mechanism of ayahuasca. An observation from the same trial suggests that higher levels of BDNF are associated with the treatment of ayahuasca and a negative correlation between BDNF and MADRS score, suggesting yet another mechanism of how ayahuasca chemically treats depression. This observation also proposed that low BDNF levels in participants with depression correlate to either very high or very low levels of cortisol. Thus, this study confirms that ayahuasca modulates cortisol levels for optimal BDNF expression, possibly contributing to neuroplasticity often elicited by psychedelic compounds [18]. In the same study, it was found that those with major depressive disorder present with significantly lower cortisol levels than the healthy group, suggesting that cortisol may play a role in depression and hormonal homeostasis. One dose of ayahuasca caused an increase in awakening salivary cortisol levels that could be attributed to the antidepressant effects of ayahuasca, in addition to its correlation with BDNF levels [20]. BDNF plays multiple roles in the peripheral and central nervous system and perhaps the modulation of cortisol via ayahuasca use proves an effective route to better understand the efficacy and biochemical qualities of this traditional brew.

Mystical experiences: There is no definitive definition of mystical experience, however, it is important to understand the general concept when looking at the effects and mechanisms by which ayahuasca is used to treat depression. The mystical experience is the intangible portion of understanding the unique mechanism by which ayahuasca can be used as a rapid antidepressant. The mystical experience is the ultimate intangible experience of unity with all things and a characteristic of being lived as an insight or state of understanding, transience, and a sense of passivity. The mystical experience is closely related to a religious experience but does not endorse supernatural or metaphysical ideas; in which one gains a new perspective on their life. Any experience qualified as mystical is characterized as different from ordinary consciousness and promotes a strong impression of contact with a different reality. Ayahuasca is one of the best-known entheogenic beverages that induces this state [27]. It is thought that the inclusive nature of these experiences and the majorly positive outcomes are what makes ayahuasca a powerful tool in treating depression, however, its highly variable and seemingly unquantifiable nature limits its acceptability.

To further understand the power of mystical experience widely reported among ayahuasca users and how it may be used for depression treatment, it is important to explore the effect of the main psychoactive compound in ayahuasca, DMT. The mystical experience transcends reality and places ayahuasca users in a suggestive state, correlating with the biochemical mechanisms mentioned above which suggest that ayahuasca use is associated with increased neuroplasticity and neuroprotective effects at the time of use [18]. In one placebo-controlled investigation, DMT effects on brain waves are a possible way to explain the mystical experience due to its suppression of alpha and beta waves paralleled emergence of theta and delta oscillations during peak effects. These findings correlated significantly with characteristic visual effects experienced by DMT users as these waves are closely associated with REM sleep which is associated with complex visual phenomena. The increased alpha waves and decreased delta waves often seen in depressed individuals combined with the findings of this study suggest that DMT can be used as a powerful tool in the modulation and treatment of depressive episodes [23]. Furthermore, another EEG study found an increase in gamma power, waves usually associated with intense focus and problem-solving, which could also contribute to the mystical experiences felt by many ayahuasca users [22]. In multiple studies, there is a strong negative correlation between depression scores and mystical experiences for ayahuasca use. In the one clinical trial to date testing the efficacy of ayahuasca, participants were asked to quantify their mystical experiences via a Mystical Experience Questionnaire (MEQ30) which was created in 2012 for similar trials. This trial found a negative correlation between MADRS scores and the MEQ30 factor of transcendence through time and space [11].

Furthermore, in a study testing the effects of DMT in a naturalistic setting, an increase in gamma power positively correlated with the MEQ30 questionnaire given to their group of 35 participants of which six were discarded. The increase in gamma power suggests that not only does the mystical experience add to one's ayahuasca treatment, but that the commonality of the mystical experience of ayahuasca may have a deeper neurological basis that can be applied to depression treatment [22]. A longitudinal study measured mystical experience via feeling oceanic boundlessness (OB), Ego Dissolution Inventory (EDI), and numerous other questionnaires to examine the scope of the intangible ayahuasca experience. This study found a negative correlation between BDI EDI and OB scores and continued to be true for almost one year after one ayahuasca dose [8]. In a cross-sectional study with 11,912 participants, many attributed affective symptom improvements to subjective mystical experiences and several personal psychological insights. Using the Short Index of Mystical Orientation (SIMO), it was seen that those with affective disorders and those who did not report affective disorders showed similar SIMO scores, with those with depression reporting greater insights during ayahuasca use. This study concluded that a greater number of mystical experiences and personal insights correlated with higher mental health outcomes. The degree of mystical experience also correlated with those reporting complete or almost complete improvement [25]. The individual nature of the mystical experience associated with ayahuasca suggests that ayahuasca is a highly individual-based treatment and can be used to treat those with moderate to severe depression effectively and rapidly.

Anxiety 

Anxiety is a disorder that affects a large portion of the global population, and it is usually accompanied by comorbidities that can make it more challenging to get to the root of the problem and treat it. Currently, numerous therapies and drugs help to alleviate the symptoms caused by anxiety, but they are not always effective in all patients and can cause significant adverse side effects [28]. New research is being done about the use of psychedelics, including ayahuasca, to treat anxiety. Ayahuasca has been shown to have anxiolytic properties and the efficacy of using it in humans is now being evaluated. Much of the research is preliminary though and produces inconclusive results as to whether or not ayahuasca improves anxiety symptoms. Most of the research that has been done discusses the rates of anxiety in long-term users or are smaller case studies, therefore more studies must be done in a controlled setting to show that it does have anxiolytic properties. Another question that has risen is the value of using ayahuasca as an anxiolytic when there are reports that one of the significant side effects includes it inducing anxiety. This discussion will further elaborate on the topics of (1) anxiety levels in regular ayahuasca users, (2) the efficacy of its use in anxiety treatment, and (3) the negative reactions to its use in treating anxiety based on an analysis of the articles.

Anxiety levels in regular users: One of the more conclusive results in studies involving ayahuasca and anxiety is its effect on regular users compared to non-regular users. One study done via survey assessed many categories of mental health, including depression, anxiety, and drug use, in regular users of ayahuasca compared to non-regular users in the same communities. Regarding anxiety, they found that those who were regularly using ayahuasca showed lower levels of anxiety [29]. Another study focused on the long-term effects on the mental health of regular participants in ayahuasca ceremonies and found similar results. Those who were regular participants reported lower levels of various mental health disorders, including anxiety, as well as better overall health and relationships with themselves and those in their communities. Additionally, the study assessed adolescents who were also regular consumers and found that, compared to other individuals of the same age, they had an improved self-perception and were more cognizant of their emotions [30]. Overall, the studies provide evidence that regular use of ayahuasca could be beneficial to mental health long term.

Efficacy in anxiety treatment: The results of many of the ayahuasca studies regarding anxiety do not provide conclusive evidence to show that it would be an effective treatment for anxiety. While some studies do show some reduction in symptoms, others report that there was not a significant difference in the level of anxiety long term. The first study was the largest of the studies that have been done and looked at the effect ayahuasca has on anxiety and depression. Regarding anxiety, it found that a significant number of participants reported some level of decrease in symptoms. This study also found a positive correlation between the level of “mystic experience” the participant experienced and the reduction of anxiety symptoms, reporting that those with more improvement typically had a more pronounced level of perceived mysticism [25]. Another study focusing on the reduction of depression and anxiety in drug users was completed by participants in ayahuasca ceremonies performed in tandem with conventional therapies. They found that there was a reduction in anxiety symptoms that was associated with an increase in self-perception [31]. This increase in self-perception, as opposed to an outward reduction in anxiety, was also seen in a study done on those with social anxiety disorder. This study found that participation in the ayahuasca ceremony greatly improved their self-perception and therefore they were able to perform the tasks better, which provided inconclusive results as to whether their overall anxiety was reduced as a direct result of the ayahuasca [28]. Additional studies focused on the subacute reduction in anxiety in those with a diagnosed anxiety disorder and found somewhat inconclusive results. Some participants reported an increase in anxiety post-ceremony but then found it decreased to below their normal levels in the following weeks while others reported that there was no reduction in symptoms. This study goes on to suggest that the use of ayahuasca might be beneficial in reducing symptoms for up to 4 weeks in non-regular users [32].

In summary, some studies are claiming that it could be useful as an anxiolytic, but more work must be done to confirm this assessment.

Negative reactions: While some studies have shown that ayahuasca might be useful in treating certain mental disorders, such as anxiety, it also produces side effects that might be counterproductive to treatment. Because of its nature as a psychedelic, it can cause dysphoric and anxiety-like reactions causing questions about its usefulness in treating anxiety if it can cause anxiety. Many studies in both clinical settings, as well as case studies, have reported participants experiencing such reactions. One study focused on the effects after the administration of ayahuasca in both patients with diagnosed anxiety disorders and those without and found that their participants experienced distressing anxiety-like reactions after administration. Additionally, they found that out of all the adverse effects experienced by participants, anxiety-like reactions were the most common [33]. Another study specifically looking at social anxiety also reported some participants had adverse anxiety-like reactions and had to be calmed down throughout the process [28]. Neither study discussed the lasting effects after administration. A final case study showed that it can produce lasting effects. A patient with generalized anxiety disorder reported increased anxiety-like symptoms for weeks after participating in an ayahuasca ceremony that required her to seek other medical interventions. Although they reported that this is an extremely rare complication, they still suggested that ayahuasca should be used with caution in those with anxiety disorders to prevent exacerbating their symptoms further [34]. While there are some perceived benefits to using ayahuasca to treat anxiety disorders, some studies have shown that this should be done with caution. Ultimately, more studies should be done to determine if the benefits outweigh the negatives. All of the studies' findings are summarized in Table 2 below. 

**Table 2:** Methods and administration of studies for anti-anxiety effects

Study	# of doses	Method of administration	Length of study	# of people	Scales used	Result
Dos Santos RG et al. (2021) [28]	1 dose	Single batch provided by the Santo Daime church Rainha do Céu, participants drank contents of vial B. caapi + P. viridis	21 days; data collected during an experimental session and during follow-up on days 7, 14, and 21	17	1^o^ outcome: Self-statements During Public Speaking Scale; 2^o ^outcomes: Visual Analog Mood Scale; Bodily Symptoms Scale, Recognition of Emotions in Facial Expressions (REFE)	Ayahuasca use had a positive impact on patients with Social Anxiety Disorder
Daldegan-Bueno D et al. (2022) [29]	Variable, self-reported, only participants with more than 1 experience included	Variable, self-reported	30-minute online questionnaire	286	WHO Quality of Life-Brief (WHOQOL-brief), Satisfaction with Life Scale (SWLS), Positive and Negative Affect Schedule-Based Questionnaire, Hospital Anxiety and Depression Scale (HAD-S), Intrinsic Religiosity Questionnaire (IRI)	Regular use of ayahuasca led to “lower anxiety (B: −0.97), negative affect (B: −2.62), general (B: 0.22) and physical (B: 0.17) quality of life, higher intrinsic religiosity scores (B: 4.16), and less past-month licit (OR = 0.30) and illicit (OR = 0.49) use of substances”
Van Oorsouw KI et al. (2021) [30]	1 dose	Participants drank ayahuasca brew prepared by shamans	4 weeks	73	Depression, Anxiety, and Stress Scale-21 (DASS-21), Brief Symptom Inventory-18 (BSI-18), Five Facets Mindfulness Questionnaire 15 (FFMQ-15), Satisfaction with Life Scale (SWLS), Ego Dissolution Inventory (EDI), 5D-ASC (Five Dimensional Altered States of Consciousness)	Participants reported decreased stress and anxiety after using the ayahuasca but no difference in level of depression
Sarris J et al. (2021) [25]	Variable, self-reported	Variable, self-reported	2017-2020	11912; 1125 with anxiety	Kessler-10 Psychological Distress Scale, Patient Health Questionnaire for Depression and Anxiety (PHQ-4)	“90% reporting an improvement in symptoms either ‘a bit’ (20%), ‘very much’ (54%), or ‘completely improved/resolved’ (16%)”
Giovannetti C et al. (2020) [31]	Variable, average of 14 sessions	Variable	January 2016 - January 2018	31	Beck Anxiety Inventory (BAI), Beck Depression Inventory (BDI)	Patients reported a decrease in anxiety and depression and an increase in quality of life and spirituality
Rocha JM et al. (2022) [33]	1 dose	Ayahuasca (1ml/kg) prepared by the Santo Daime church Rainha do Céu	6 months	17 volunteers with social anxiety disorder	Psychological Adverse Reactions (PARs)	Some patients experienced psychological adverse reactions including fear, distress, anxiety, and dissociation
		Ayahuasca (1mL/kg) prepared by the Santo Daime church Rainha do Céu + cannabidiol (CBD, 600 mg)		17 healthy volunteers		
dos Santos RG et al. (2017) [34]	2 doses	Ingestion, self-reported	Case report	1	N/A	Patient with diagnosed generalized anxiety disorder reported extremely heightened anxiety and panic after participating in an ayahuasca ceremony

Use of Ayahuasca in the Management of Substance Abuse and Addiction

A consistent body of research suggests that ayahuasca has promising therapeutic applications for substance abuse and addiction, particularly concerning substances such as alcohol and tobacco [35]. Various psychometric assessments have allowed researchers to develop an understanding of the dynamic interplay between ayahuasca’s effects and participants’ urges to consume alcohol or tobacco products. Results from various self-report questionnaires, such as the Questionnaire on Smoking Urges-Brief (QSU-B) conducted by Daldegan-Bueno et al., and the Substance Abuse and Mental Health Services Assessment (SAMHSA) conducted by Barbosa et al., revealed that individuals who had tobacco addictions before attending an ayahuasca ceremony reported diminished cravings after each subsequent ceremony [35,36]. However, it is hypothesized that there is a strong interdependence between ayahuasca consumption and its religious context. Meaning that using ayahuasca outside of the religious context, and religious intervention devoid of ayahuasca supplementation exhibit limited therapeutic efficacy as it relates to alcohol and tobacco use disorders. Perkins et al. emphasize this point by suggesting that the religious environment in which ayahuasca is typically consumed could potentially help offer therapeutic benefits distinct from the effects of ayahuasca alone [37]. Furthermore, the studies that focused exclusively on ayahuasca consumption within religious environments such as the ceremonies hosted by the União do Vegetal (UDV) revealed that a majority of participants exposed to ayahuasca in these traditional settings had a significant reduction in alcohol and tobacco use compared to the Brazilian norm [35,37] while the broader studies, that included global participants from countries without historical or religious ayahuasca use, as well as recreational users of ayahuasca provided mixed results in regards to treating substance abuse and addiction [38]. Suggesting that ayahuasca consumption outside of a religious context did not facilitate cessation to the same degree. Thus, supporting the hypothesis that the religious ceremony together with the consumption of ayahuasca is necessary to provide effective therapeutic benefits that either help users reduce or completely cease the use of alcohol or tobacco products [37]. To further investigate this point, this subtopic will be divided into three main categories that focus on the following: (1) comparing the therapeutic effects of ayahuasca with other psychedelics, (2) the significance of the ritualistic practices and social setting of the ritual, and finally, (3) the mystical experience as it relates to assessing the effects of therapy. These above-mentioned studies’ findings are summarized in Table 3 below.

**Table 3:** Methods and administration of studies for the management of substance abuse and addiction

Study	# of doses	Method of administration	Length of study	# of people	Scales used	Result
Barbosa PC et al. (2018) [35]	Variable, self-reported	Ingestion, self-reported	The study was conducted between March 2009 and August 2011, approximately 2 years and 5 months	1,947	The study used questionnaires developed by the World Health Organization (WHO) and the Substance Abuse and Mental Health Services Administration (SAMHSA) to assess alcohol and tobacco use	Regression analyses showed that attendance at ayahuasca ceremonies during the previous 12 months and years of União do Vegetal (UDV) membership significantly impacted the reduction of alcohol and tobacco use disorder
Daldegan-Bueno D et al. (2022) [36]	Variable, self-reported	Ingestion, self-reported	The study used a retrospective cross-sectional design, meaning it analyzes data collected at a specific point in time from an online survey. The exact duration of the study is not specified	441	Questionnaire on Smoking Urges—Brief (QSU‐B), Fagerström Test for Cigarette Dependence (FTCD) Mystical Experience Questionnaire (MEQ30)	The study found that mystical experience (measured by MEQ30) and the frequency of ayahuasca intake were protective factors associated with smoking cessation. Additionally. positive mood during the ayahuasca experience was identified as a risk factor for smoking cessation
Perkins D et al. (2022) [37]	Variable, self-reported	Ingestion, self-reported	The data for the study were collected between April 2017 and May 2019, spanning approximately 2 years	8,629	Questionnaires and self-reported data	The study found a strong positive association between the number of times ayahuasca had been consumed and decreased odds of drinking alcohol, engaging in risky drinking and using a range of drugs in the past month. These effects were more pronounced in individuals with prior substance use disorders
Lawn W et al. (2017) [38]	Variable, self-reported	Ingestion, self-reported	The study conducted an online, self-selecting, global survey examining drug use patterns in 2015 and 2016	96,901	Kessler-10 Psychological Distress Scale, Patient Health Questionnaire for Depression and Anxiety (PHQ-4)	Ayahuasca users reported greater well-being than classic psychedelic users and non-psychedelic drug users

Psychedelic therapy for addiction: ayahuasca vs other psychedelics: There is a growing interest in the therapeutic applications of psychedelics for treating addiction and substance abuse. A study conducted by Lawn et al. compared the therapeutic potential of ayahuasca to other classic psychedelics such as LSD and magic mushrooms [38]. In this international cross-sectional survey, Lawn et al. compared current and lifetime drug use amongst three distinct demographic groups [38]. The first group consisted of past-year ayahuasca users (i.e., users who have only consumed ayahuasca and no other psychedelics in the past year), the second group consisted of past-year LSD and magic mushrooms users (i.e., classic psychedelic users who have not consumed ayahuasca previously), and lastly, the third group consisted of participants who have not consumed ayahuasca or any other psychedelic drugs in the past year. A total of 96,901 participants were included in this study. Of these participants 527 participants were included in the ayahuasca group, 18,138 were included in the classic psychedelic user group, and 78,236 were included in the non-psychedelic user group. All participants completed the Global Drug Survey (GDS). Based on the survey results from the Global Drug Survey (GDS), it was determined that both the ayahuasca users and classical psychedelic users reported a reduction in their current tobacco and alcohol consumption after exposure to their respective psychedelics in the past year. The ayahuasca users reported a significantly greater reduction when compared to users of LSD and magic mushrooms. This suggests that ayahuasca has more therapeutic potential as it pertains to substance abuse and addiction than classical psychedelics. The GDS also assessed the participant's physical and psychological experiences while exposed to ayahuasca or classical psychedelics.

Interestingly, the results revealed that the users of ayahuasca generally rated their experiences as “stronger,” “less pleasurable,” and having “more negative side effects'' compared to the classic psychedelic users. Indicating that the psychoactive effects of ayahuasca lead to distinct physical symptoms compared to classic psychedelics like LSD and magic mushrooms [38]. The generalized report of ayahuasca, being “less pleasurable” and having “more negative effects,” may contribute to its greater therapeutic effects. This idea is supported by a study conducted by Daldegan-Bueno et al., which explained how ayahuasca helps reveal repressed emotions that are the root of negative self-beliefs and addictive behaviors [36]. The reported symptoms that aided in tobacco cessation were divided into eight common themes, five of which were related to the characteristics of the ayahuasca experiences that led to successful smoking cessation or reduction, (1) acquired awareness, (2) sensorial experiences, (3) purging, (4) spiritual experiences, and (5) non-specific or inexplicable experiences. These symptomatic experiences were correlated with a decreased desire to consume tobacco [35]. Furthermore, the ayahuasca user may begin to create an association between their addictive pattern and the negative aspects of their ayahuasca experience, especially the vomit-inducing effects of ayahuasca [37]. Conversely, Daldegan-Bueno et al. also provide evidence to suggest that a “positive mood,” while experiencing the effects of ayahuasca, measured by the ​​mystical experience (MEQ30) is a risk factor for ineffective treatment [36]. Further emphasizing the importance of the uniquely unpleasant experience of ayahuasca exposure [37].

These results and claims proposed by Daldegan-Bueno et al. help to interpret why participants in the Lawn et al. study, who reported the generally “less pleasurable” experience of ayahuasca were more likely to reduce their substance use compared to classical psychedelic users who reported a relatively more pleasurable experience [36,38]. A limitation of this broad study conducted by Lawn et al. is that it did not exclude ayahuasca use outside of the Brazilian religious ceremony since it includes global recreation users of ayahuasca in the study [38]. As a result, when compared to the non-psychedelic group, these global and largely recreational users of ayahuasca demonstrate a far lesser reduction in alcohol and tobacco use [38]. The study suggested that the inclusion of participants who consumed ayahuasca outside of the religious context may be responsible for the diminished therapeutic results [38]. For example, a study conducted by Barbosa et al. focused exclusively on respondents who were primarily from specific regions of Brazil where ayahuasca is largely controlled by religious organizations such as the UDV [35]. The differences in these two studies' results could explain the importance of consuming ayahuasca in a religious context, as the Lawn et al. study included global participants who used ayahuasca independently, which might explain why they did not reap the benefits obtained by those who consumed ayahuasca [38]. The results from the Lawn et al. study suggest that compared to other psychedelic drug users, ayahuasca users had lower rates of alcohol and tobacco consumption [38]. This evidence indicates that the effects of ayahuasca are not simply due to the hallucinogenic state of mind that all psychedelics induce but are more likely a result of the unique effects and psychogenic properties of the ayahuasca itself. Furthermore, the inclusion of recreational users of ayahuasca suggests that the religious environment, such as the one provided by the UDV is essential for achieving the full therapeutic potential of ayahuasca.

The significance of the ritual and the social setting: It has been hypothesized that the religious environment of the ayahuasca ceremony in tandem with ayahuasca itself is necessary for achieving the full therapeutic potential of ayahuasca. In the study conducted by Barbosa et al., a cross-sectional design was used to survey 1,947 UDV volunteers [35]. Participants were surveyed using the SAMHSA questionnaire to determine the presence of a substance use disorder among these participants. Furthermore, the participants were also surveyed with a modified version of the alcohol and tobacco sections of the WHO Research and Reporting Project on the Epidemiology of Drug Dependence questionnaire, and results from these surveys were used to determine alcohol and tobacco use before and after joining the UDV. Finally, these results were compared to the findings of a standardized survey conducted by the Centro Brasileiro de Informações sobre Drogas Psicotrópicas (CEBRID), which included 7,939 randomly selected subjects from Brazil to estimate the norm of substance use among the general Brazilian population [35].

The results from the Barbosa et al. study revealed that the UDV members who were ages 25 to 34 had a significantly greater lifetime exposure to alcohol and tobacco before joining the UDV compared to the Brazilian norm [35]. Meaning, that before joining the UDV these participants generally consumed more alcohol and tobacco products than the standard Brazilian adult. However, the result also revealed that after their first exposure to ayahuasca in a UDV ceremony, these participants reported a significantly lower prevalence of alcohol and tobacco consumption when compared once again to the Brazilian norm [34]. The decreased levels of alcohol and tobacco consumption were positively correlated with years of attendance as the participants who had more than 3 years of attendance with the UDV had a reduction of substance use of a far greater magnitude. These results are noteworthy as they indicate that these UDV members who previously consumed more alcohol and tobacco than the general Brazilian population, currently consume far less than the general Brazilian population does, emphasizing the potential therapeutic benefits of ayahuasca in managing substance abuse [35].

Determining whether the reduction in alcohol and tobacco consumption among these UDV members is a result of ayahuasca consumption or ayahuasca consumption in tandem with the religious rituals and setting of the UDV is made clear by the results of the Lawn et al. study that did not limit their survey to religious users of ayahuasca [38]. Similarly to the Barbosa et al. study, the global participants of the Lawn et al. study also reported greater lifetime exposure to alcohol and tobacco [35,38]. These global participants also reported reduced consumption of alcohol and tobacco after their exposure to ayahuasca. However, these participants still maintained higher consumption of alcohol and tobacco compared to the control, despite their current use of ayahuasca [38].

These results help to support the idea that the religious setting and rituals such as those provided by the UDV are important therapeutic factors regarding managing substance use and addiction [35,38].

The mystical experience and other psychoactive factors as they relate to substance use therapy: A study conducted by Daldegan-Bueno et al. surveyed 441 former tobacco smokers who had either reduced or completely quit their cigarette consumption after at least one ayahuasca experience in a religious setting [36]. Unlike other studies included in this subtopic, this study conducted by Daldegan-Bueno et al. focused exclusively on individuals who previously had a dependency on tobacco but successfully experienced the therapeutic effects of ayahuasca [36]. Of the 441 participants 305 completely quit smoking, 81 reduced their consumption and 55 quit but later relapsed. Interestingly the majority of the participants in this study reported that they did not intend to cease or reduce their consumption of cigarettes. This may help to contradict any claims that ayahuasca is acting as a placebo. Furthermore, this study used three distinct qualitative assessments to analyze the common themes and characteristics related to the reduction and quitting of tobacco in these participants. The eight themes were: (1) acquired awareness, (2) sensorial experience, (3) purging, (4) spiritual experience, (5) inexplicable experiences, (6) repulsion to cigarettes, (7) immediate or gradual cessation, and (8) increased motivation. Based on the questionnaire completed by participants, one or more of these themes may have arrived during exposure to ayahuasca facilitated by the mystical experience that accompanies the use of the drug [36]. Several studies emphasize the importance of the mystical experience measured by the MEQ30 mystical experience questionnaire, as the effects of the mystical experience help shed light on the root of the addictive behaviors thus aiding in cessation. In addition, The score obtained from the MEQ30 questionnaire is a valuable predictor of smoking cessation amongst different demographic groups, as the mystical experience is also understood to be responsible for the alleviation of psychological symptoms associated with tobacco dependency such as anxiety and depression [36,37]. In addition to their surveys and assessments administered in this study, Deldegan et al. also performed a 12-month follow-up where there was found to be a negative correlation between the MEQ30 scores and urine nicotine levels after 12 months of ayahuasca exposure [36].

Overall, the results from this study help explain the significance of the mystical experience and how it contributes to smoking cessation. The results also suggest that there are lasting long-term effects, but further investigation is needed to understand the long-term implications of ayahuasca exposure [36].

Eating Disorders

Eating disorders encompass a wide range of disorders that involve an unhealthy relationship to food and/or weight loss, affecting the patient's physical and mental well-being [39]. Eating disorders, including anorexia nervosa, bulimia nervosa, and binge-eating disorder, are commonly associated with comorbidities that either prefaced the development of an eating disorder or followed its development. These comorbidities are also typically psychiatric, such as depression, anxiety, and substance abuse, and contribute to the difficult nature of treating eating disorders. Patients with eating disorders often experience high rates of incomplete treatment and therefore high relapse rates [39]. Because of the lower success rates of conventional, Western treatments, many studies are being done to assess the usefulness of other treatment options, including the use of ayahuasca. While most of the studies involved interviewing participants after completion of the ceremony, one study was also done by interviewing ayahuasca ceremony leaders on their perceived value of the ceremony in treating eating disorders. Ceremony leaders with varying educational backgrounds and years of experience working with ayahuasca reported on their ideas surrounding eating disorders and how they develop as well as the perceived benefit that participants with eating disorders receive by completing the ayahuasca ceremony [39]. Further studies have assessed the level of symptom reduction in participants with eating disorders after completing a ceremony as well as their recommendations on its usefulness. These studies have typically been done on smaller cohorts and often include participants who were not successful with conventional therapies. One study commented on the psychological, physical, and contextual aspects of healing gained via the ceremony and reported a significant reduction or elimination of eating disorder symptoms in a majority of the participants due to the self-revelations obtained from the ceremony [40]. A similar study focused on the emotional and spiritual aspects of healing from an eating disorder and found similar results of reduced or eliminated symptoms of both the eating disorder and the other comorbid conditions, as well as an increase in the emotional competency and evaluation of self-worth [41]. Topics that will be discussed further include (1) the perceived methods by which ayahuasca treats eating disorders, (2) the concerns involving the required preparatory diet and cleansing of the ceremony, and (3) the lasting effects of the ceremony on the reduction or elimination of eating disorder symptoms based on an analysis of the articles mentioned in Table 4.

**Table 4:** Methods and administration of studies for effect on eating disorders

Study	# of doses	Method of administration	Length of study	# of people	Result
Williams M et al. (2023) [39]	Variable, 2-20 years of experience working with ayahuasca	Variable, self-reported	90-minute interview	15 ceremony leaders; 10 of whom had experience with EDs	Leaders described theories about eating disorders (EDs) as “symptomatic of an underlying concern,” as “serve a function,” and “affect health in multiple domains” Leaders described theories about how ayahuasca affects individuals with EDs as “facilitate ‘energetic healing," as “helps identify, process, and integrate the ‘root’ of the ED,” as “promotes holistic healing,” and “enhances and/or reorganizes relationships”
Lafrance A et al. (2017) [40]	Variable, between 1-30 ceremony participations	Variable, self-reported	75-180 minute interview	16; 10 with anorexia nervosa, 6 with bulimia nervosa	Most participants reported a decrease in ED psychological symptoms, a generally positive approach to body perception and physical sensation, the importance of having a safe space for healing
Renelli M et al. (2020) [41]	Variable, between 1-30 ceremony participations	Variable, self-reported	120-minute interview	13; 8 with anorexia nervosa, 5 with bulimia nervosa	Participants reported that ayahuasca use “(1) was more effective (2) allowed for deeper healing (3) allowed for the processing of intense emotions and/or memories, (4) provided lessons in and discoveries of love, self-love, and self-care, and (5) provided a spiritual component to healing and recovery”
Spriggs MJ et al. (2021) [42]	Variable	Variable, self-reported	3-4 weeks; data collected 1-2 weeks before, 2 weeks after	28 complete baseline and post-experience assessment; 27 completed acute experience assessment	The use of ayahuasca promoted “positive psychological aftereffects of a psychedelic experience” in patients who had been diagnosed with EDs

Methods of healing: While there are many proposed theories as to how ayahuasca can be used to treat eating disorders, there are two main ones discussed in many of the studies that have been done. The first centers around getting to the “root” of the eating disorder. Several studies discussed the importance of this in beginning the journey to healing and reducing symptoms. They reported that the ayahuasca helped participants by “bypassing the individual’s defense mechanisms” allowing them to start from the beginning in a sense to reframe their thoughts and self-perceptions [39]. By allowing themselves to journey into the deepest layer of themself, they can work towards building up and resolving what is at the root of the issue through further self-assessment and treatment [41]. Other studies focused on getting to the root of the problem to shift their negative self-perceptions, whether that be blame, self-worth, body image, or shame, into something that is more productive and improves their self-worth. These ideas lead to the second main theory of how ayahuasca can be beneficial in treating eating disorders. This second theory revolved around the idea that the ceremony can help to recategorize the participants' relationships with themselves and their eating disorders. Treating the emotional aspects of eating disorders has been proven to play a large role in the successful reduction or elimination of symptoms. According to studies, the ceremonial use of ayahuasca has helped participants to “recontextualize” the impact their eating disorder has had on their lives and let go of the negative emotions that surround it. It allows them to reinvent their connection with their body and frame it in a more self-loving and compassionate way [40].

Participants overall reported that the ability to “process unresolved emotions such as grief and shame” brought to the surface during the ceremony allowed for a new starting point in their treatment and healing journey by allowing them to make choices that were better for themselves [41]. Another study showed that this emotional release and reconnection played a large role in the long-term success of the eating disorder treatment [42]. These two main theories of how ayahuasca affects the reduction of eating disorder symptoms work together as participants get to the root of their disorder and then use that to reassess their emotional relationship to themselves and their eating disorder, leading toward more effective healing. 

Concerns about preparatory diet and cleansing of ceremony: Participation in the ayahuasca ceremony can be very cathartic but it involves a ritualized preparatory diet involving many food restrictions before the ceremony begins and then the ceremony can begin, then participants will go through a cleansing throughout the ceremony that involves purging/vomiting [40]. Considering dieting and purging are common symptoms of many eating disorders, this brings to question the efficacy of a treatment that puts these behaviors at the forefront. Two different perspectives were assessed in various studies to understand how the ceremony and its components are framed from a ceremony leader's point of view and to understand how the participants with eating disorders cope with these behaviors being integrated into their treatment. From the perspective of a ceremony leader, studies have shown that this is a topic of conversation, as they do not want to encourage already present eating disorder symptoms. The leaders have found that putting the negative behaviors in the context of “energetic cleansing,” can reframe the participants' relationship with their eating disorder and allow the behaviors to become more positive in the context of the ceremony [39]. From the perspective of the participant, studies have reported a similar finding. When the behaviors of dieting and purging were put into the context of the ceremony, all the participants were able to separate the purging due to their eating disorder and the purging in the ceremony, avoiding “feeling triggered or symptomatic” [40]. Another study reported that the purging due to their eating disorder was a very different experience from the purging during the ceremony and it allowed for freeing of the negative feelings and emotions that have been holding back their healing process [39]. While dieting and purging/vomiting are key symptoms of many eating disorders, many studies evaluating the perception of these behaviors from both a leader's perspective and a participant's perspective have shown that there is no exacerbation of symptoms due to the ceremony.

Lasting effects of treatment with ayahuasca: All the studies have shown promising evidence that the ayahuasca ceremony can provide lasting reduction or elimination of eating disorder symptoms. The effects were often experienced long-term over months or years and some even reported a lack of symptoms not relapsing back into the eating disorder. Similar to most treatments, there is a varying level of success in the reduction of symptoms with the treatment. Those who experienced elimination of symptoms reported that “it was like my brain was reprogrammed” as the ceremony helped to rewire their negative thoughts and eliminate the compulsory need to give in to the urges or symptoms precipitated by their eating disorder. Additionally, most participants, regardless of the level of symptom reduction, reported an improved ability to process their emotions and increased self-love, leading to subsequent success in additional treatment after using ayahuasca [40]. Other studies reported similar findings but focused on comparing the success participants found after conventional treatments to after completing the ayahuasca ceremony. They found that a majority of the participants reported that their perception of symptom reduction was better after using ayahuasca compared to their experiences with conventional therapies [41]. The studies concluded their assessment of the efficacy of ayahuasca use on the treatment of eating disorders with recommendations on how to proceed, stating the importance of current conventional therapies and how ayahuasca could potentially be integrated to form more complete and holistic treatment options for those struggling with an eating disorder [40,41].

Use of Ayahuasca in the Management of PTSD and Childhood Trauma

Studies investigating the potential therapeutic application of ayahuasca for the management of PTSD and past trauma are limited. The psychological effects of PTSD and other trauma-related mental illnesses are complex and differ immensely from person to person. The majority of the research conducted under this subtopic investigates the effects of ayahuasca on fear and PTSD via animal studies or in vitro models. Therefore, most of the evidence supporting the use of ayahuasca for these trauma-related mental disorders consists largely of anecdotal reports and theoretical hypotheses. However, the available human studies conducted within this subtopic suggest that the potential therapeutic applications of ayahuasca, as it relates to the treatment of PTSD and childhood trauma, have lasting biochemical effects that promote synaptic neuroplasticity, as well as memory retrieval, all of which are hypothesized to facilitate the reprocessing of repressed or previously forgotten traumatic memories [19,43]. To expand on the topic of PTSD and trauma this subtopic will investigate the epigenetic effects of ayahuasca and its applications in the reprocessing of trauma, with a particular focus on childhood trauma based on an analysis of the articles mentioned in Table 5.

**Table 5:** Methods and administration of studies for effect on post-traumatic stress disorder and trauma

Study	# of doses	Method of administration	Length of study	# of people	Scales used	Result
Ruffell SG et al. (2021) [43]	Variable, self-reported	Ingestion of approximately 150 ml of the prepared ayahuasca brew. The brew was administered to participants in ceremonies lasting around 5 hours each. The brew was prepared and provided by a local curandero (shaman). Participants were instructed to set intentions before the ceremonies and consumed the medicine in a ceremonial context.	The study involved pre-retreat measures, post-retreat measures, and a follow-up 6 months after the final ceremony	63	Beck Depression Inventory-Second Edition (BDI-II), State-Trait Anxiety Inventory (STAI), Self-Compassion Scale (SCS), Clinical Outcomes in Routine Evaluation-Outcome Measure (CORE-OM), Childhood Trauma Questionnaire (CTQ), Mystical Experience Questionnaire (MEQ), and Sentence Completion for Events From the Past Test (SCEPT)	The study reported a statistically significant decrease in BDI-II scores, STAI scores, and CORE-OM scores post-retreat, along with an increase in SCS scores. These improvements in mental health measures were sustained at the 6-month follow-up, suggesting potential lasting therapeutic effects
Inserra A (2018) [19]	--	--	--	--	--	The passage discusses a hypothesis regarding the potential effects of ayahuasca on traumatic memories and suggests that further studies are needed to investigate these effects

Epigenetic effects of PTSD and childhood trauma: An observational naturalistic study conducted by Ruffell et al. surveyed 63 self-selected attendants of a Peruvian ayahuasca retreat [43]. The study included various surveys, including an assessment of childhood trauma using the Childhood Trauma Questionnaire (CTQ). It also included an epigenetic evaluation of the participants. This study is significant as it is the first to explore the epigenetic involvement of ayahuasca with the SIGMA-1 receptor in human subjects [43]. SIGMAR1 is understood to promote cell survival, neuroprotection, neuroplasticity, and neuroimmunomodulation. Therefore, ayahuasca's involvement with this receptor could potentially be used as a pharmaceutical treatment that helps reverse the neurogenetic consequences of trauma [19]. To assess the epigenetic function of ayahuasca in individuals struggling with trauma, the changes in DNA methylation patterns were evaluated by evaluation of participants' saliva before and after exposure to ayahuasca. While the function of ayahuasca on the SIGMAR1 is still poorly understood, this investigation provided data to support the use of ayahuasca for the treatment of trauma [43].

Within the study conducted by Ruffel et al., a modest correlation was found between childhood trauma and SIGMAR1 methylation changes [43]. Furthermore, the study employed the CTQ to assess distinct types of childhood maltreatment before ayahuasca exposure. Notably, participants with higher CTQ scores exhibited increased epigenetic methylation of the SIGMAR1 receptor after ayahuasca exposure, indicating an epigenetic interaction between ayahuasca and SIGMAR1. This correlation supports a potential link between trauma and SIGMAR1’s epigenetic regulation [43]. SIGMAR1 induces anti-amnesic effects allowing the repressed memories to be retrieved. PTSD is characterized by repression, so the anti-amnesic effects of ayahuasca allow the origin of the fear associated with the traumatic event to be retrieved and reprocessed. The exact mechanism behind the reprocessing of these traumatic memories is unclear. However, SIGMAR1 is known to boost synaptic plasticity which is an effect that may be responsible for the reported healing in ayahuasca participants struggling with PTSD [19]. The strength of this correlation between ayahuasca and SIGMAR1 methylation, as provided by the Ruffel et al. study, is limited [43]. However, the correlation found by this study reveals how trauma-related experiences influence molecular pathways tied to SIGMAR1 expression. Supporting the potential pharmaceutical application of ayahuasca for PTSD and trauma treatment. However, the biological implications of these results warrant further investigation [19,43].

Overall, participants experienced improved mental health outcomes following ayahuasca use in a traditional Amazonian setting. These sustained positive changes prompt inquiries into SIGMAR1's potential role in mediating ayahuasca's therapeutic effects. Considering SIGMAR1's involvement in stress response, neuroprotection, and neuroplasticity, it is plausible that the receptor plays a substantial role in the observed mental health enhancements. These findings lay the groundwork for future research into the molecular mechanisms of ayahuasca's impact on PTSD and childhood trauma, particularly focusing on SIGMAR1 and its epigenetic modulation.

*Personality Changes* 

All studies used in this subtopic which are mentioned in Table 6 are longitudinal and followed up with participants multiple times post-ayahuasca use [44]. Each of them has a considerable effect on individual personality. The following subtopic will be divided into influence on cognitive thinking, mindfulness, and ego dissolution. Multiple studies explain the observed personality through personality models including the Five Factor Model. A study sampling 256 participants proposed that non-ordinary states of unitive consciousness, insightfulness, and mystical experience were likely to increase Openness and Extraversion while decreasing neuroticism post-ayahuasca use. Comparably participants with higher levels of neuroticism had a larger adaptive change post-ayahuasca use. Moreover, the study showed that the changes in neuroticism post attending an average of 4.4 ayahuasca ceremonies were proportional to effects seen after multiple clinical interventions. On the other hand, the study’s support for Openness and Extraversion was weak, which was explained as a possible ceiling effect. Consistent with reports and analysis of ayahuasca’s non-pharmaceutical effects, mystical experience did indeed show a correlation with the highest personality change. The mystical experience was described as finally being part of something larger, developing self-love and acceptance. These psychological characteristics have been described in multiple models including Freud's “oceanic feeling” and Maslow’s “peak experience”. This strong evidence of ayahuasca’s effects correlating with accepted psychological models continues to add more to the rationality of exploring it as a therapeutic potential. While the above study does provide support to multiple themes of personality change the data is only self-reports [44]. Hence, no placebo groups or controls were possible. This is a consistent limitation seen in most studies used in this subtopic. Table 6 summarizes all the above-mentioned studies’ findings.

**Table 6:** Methods and administration of studies for personality changes

Study	# of doses	Method of administration	Length of study	Personality trait model used	# of people	Result
Weiss B et al. (2021) [44]	1	Self-report at three stages: Baseline, Post, 3 months follow up	3 months	Five Factor Model	256	Post 3 months the greatest decrease in Neuroticism both self-report and informant reported. Extravasation and Openness did not change despite the original expectation. The degree of the adaptive changes largely depended on the participants' baseline changes
Murphy-Beiner A et al. (2020) [45]	1	Self-report before and after ceremony	24 hours	Five Facets Mindfulness Questionnaire (FFMQ), Experiences Questionnaire (EQ), Cognitive Flexibility Scale (CFS), Stroop and Wisconsin Picture Card Sorting Task (WPCST)	48	Mindfulness with 4/5 points on FFMQ, decentering and cognitive flexibility significantly increased post ayahuasca use. This improvement was described to be part of the “afterglow period” and suggested a possible psychological mechanism.
Pasquini L et al. (2020) [46]	1	MRI imaging 1 day before and after ceremony	2 days	None	21 placebo group, 22 ayahuasca group	MRI showed an increase in activity within the salience network in the anterior cingulate cortex connectivity. In comparison, there was a decrease in connectivity in the posterior cingulate cortex connectivity within the default mode network. There was an increased connectivity between the salience and default mode networks in the ayahuasca group compared to placebo. These results coincided with an increase in emotion and self-empowerment.
Kiraga MK et al. (2021) [47]	1	Self-report: baseline, the morning after, and 1 week after	1 week	Picture Concept Test, Multifaceted Empathy Test, Satisfaction with Life Scale, Experiences Questionnaire, Big Five Inventory	43	At 7-day self reports an increase in cognitive empathy, satisfaction with life, emotional empathy, and decentering was observed in comparison to divergent thinking, neuroticism decreased
Soler J et al. (2018) [48]	4	Self-reports	8 weeks	The Five Facet Mindfulness Questionnaire (FFMQ), The Experiences Questionnaire (EQ), The MINDSENS Composite Index	10 Ayahuasca group, 10 mindfulness training programs	The mindfulness group had a greater increase in mindfulness and was the only group to have an increased MINDSENS index. Ayahuasca group did have a comparable increase in acceptance of the FFMQ, and improved detachment with decreased judgment.

Cognitive effects of ayahuasca: The 5 HT2a agonist property of ayahuasca in addition to its biochemical effects has been said to have an afterglow effect in participants for up to 2 months due to the increase in positive mood. Researchers have recently begun examining for any psychological explanation for this afterglow, cognitive flexibility being one of them. A negative correlation between decreased cognitive flexibility and psychopathology has been explained. A study with 54 participants measured cognitive flexibility pre-ayahuasca and 24 hours after use. The Cognitive Flexibility Scale (CFS), Stroop Colour and Word Task (SCWT), and Wisconsin Picture Card Sorting Task (WPCST) were used to measure cognitive flexibility. Results showed a positive increase in ratings of both CFS and WPCST testing suggesting a significant improvement in cognitive flexibility since baseline [45]. This was seen in both naive users and long-term users. Given that across depression, anxiety, and ED patients there is a general theme of fixation on thought, behavior, and pattern. The effect of ayahuasca relaxing and widening the mind can be beneficial by developing open-mindedness and acceptance of help from friends, family, and other therapeutic alternatives.

The physiological explanation of increased cognitive flexibility has been explained through the decrease in functional connectivity in the Default Mode Network (DMN, in which the medial prefrontal cortex {mPFC} and posterior cingulate cortex {PCC} are key hubs), changes in the central executive network (CEN), and increase in blood flow to the salience network (SN) (in which the anterior cingulate cortex {ACC} is involved) and posterior region excitation post-ayahuasca. All these combined are thought to ease up the frontal cortex which is involved in executive control. A recent study examining functional MRI scans before and after ayahuasca use in cognitively and physically healthy naive 50 participants in a placebo-controlled experiment provided evidence for this physiological process. There was a salience network connectivity increase within the anterior cingulate cortex and between the anterior cingulate cortex and the superior frontal gyrus in ayahuasca compared to the placebo. In addition, increased connectivity between the salience and default mode networks in the ayahuasca group was observed. Default mode network connectivity decreased within the posterior cingulate cortex in the ayahuasca group. This decrease in DMN provided evidence of the experience participants had in interacting with themselves during the ceremony. This is “decentering,” the ability for one to observe their thoughts and emotions in the third person [46].

A study with 64 participants measured the extent of decentering, creative thinking, and decentering. This was the first study to show a significant increase in participants’ ability to correctly identify and label emotions, this was defined as emotional empathy. The increase in empathy is likely to be a combination of decentering and increased cognitive flexibility. Individuals with depression, anxiety, and PTSD typically have low empathy, this new property in addition to the multiple discussed in the review further demonstrates the holistic potency of ayahuasca as a therapeutic drug [47].

Ego dissolution and mindfulness in ayahuasca participants: Ego death or dissolution in ayahuasca participants is seen because of a change in thinking styles including mindfulness which refers to an increase in awareness, observation, non-judgment of self, and non-reactivity to inner experiences. Increase in creative and divergent thinking opening participants up to different perspectives. A study with 57 participants measured the sub-acute and long-term effects ayahuasca has on ego dissolution and the positive changes associated. Subjective measures in the study included Depression and anxiety Stress scales, Satisfaction with Life scales, Five Facet Mindfulness Questionnaire, and Ego Dissolution Inventory. Results were measured at baseline, the day after, and four weeks after (in 31 participants only). Changes in effect, Satisfaction with life, and mindfulness showed association with ego dissolution. While depression, stress, and convergent thinking showed a significant decrease 24 hours after and remained low four weeks after, satisfaction with life and mindfulness did not persist significantly until four weeks. This showed that participants experience ego dissolution via mindfulness during the “acute phase” and the effects on depression and stress continue to stay in effect long term [32].

Mindfulness in itself has gained increased popularity in not only managing depression, anxiety, and stress but also overall well-being. The most common practice is Mindful Based Stress Reduction (MBSR), an eight-week-long program. The MBSR included a body scan where participants focused on isolated parts of the body, yoga to develop an awareness of the body, and sitting meditation to focus on breathing sounds and vibrations. A total of 20 participants were divided into four consecutive ayahuasca session groups or the MBSR group. To assess mindfulness participants filled out the Five Facet Mindfulness Questionnaire (FFMQ).

“Acceptance” rating of FFMQ. The parallel increase in both groups adds to the characteristics of ayahuasca. While all the participants in MBSR joined the group wanting to acquire mindfulness, participants in the ayahuasca groups engaged for reasons beyond. Ayahuasca’s ability to increase mindfulness in participants even when that was not their primary goal growing into the ritual adds to the holistic effect it has [48].

The approach ayahuasca ceremonies use appears to have much in common with Mindfulness Cognitive Behavior Therapy (MCBT), a Western psychological behavioral treatment well accepted for depression, anxiety, PTSD, and eating disorders. The goal of MBCT is to confront feelings and thoughts but in a non-judgmental manner and observe all of them as the one feels them [49]. Participants from the study observing 64 participants over days expressed decentering and increased mindfulness showing a continued presence even on the seventh day. The decentering allows participants to face and be aware of their current consciousness, they can analyze their feelings, relive autobiographical memories, and gain a perception of their problems [47]. These parallels in MCBT and ayahuasca demonstrate the effectiveness of ayahuasca as a step towards a more holistic, efficient therapy for mental illnesses. Similarly, shamans described ayahuasca as a tool for individuals with eating disorders to face their repressed and suppressed thoughts, and feelings and accept them as present even the negative [39].

Ritualistic Impacts of the Ayahuasca Ceremony

Ayahuasca is not discussed independently from the ceremony. Currently, the majority of clinical studies studying ayahuasca’s effect on mental illness include longitudinal, self-evaluations, and cross-sectional studies; all including participants using Ayahuasca in the ritualistic setting only. One can participate in an ayahuasca ritual through three main channels available globally: via Brazilian syncretic churches that use ayahuasca with a communal-religious intention and incorporate different spiritual and religious traditions such as Christianity, African, and Amazonian traditions. Second via shamanic tourism in which Western participants travel to the Amazon to experience ayahuasca ceremonies and seek psycho-spiritual growth. Third is an underground system that is worldwide, and the facilitator is called a new shaman. These neo-shamans combine indigenous practices with some forms of Western therapeutic practices and practices based on their own identity, or local to their countries. These public rituals have been emphasized and continued in indigenous practices to encourage social identity creation. A qualitative research study interviewed 31 Arab Palestinians and 18 Jewish Israelis. These interviewees have established members of different local ayahuasca groups and are well experienced. The interviewees’ initial motivation to join the group was personal psycho-spiritual growth. None of the interviewees were aiming for reconciliation or peacebuilding. Like a typical ritual, music was a central component for all these groups however the singing was not limited to the shaman, everyone joined in and in some instances, participants also had the opportunity to share their music and prayers. The Interview was aimed at gaining the interviewee’s opinion on ayahuasca in an Israeli-Palestinian context. The grounded theory approach was used to analyze data [50]. 

Three themes were identified from the articles listed in Table 7: (1) Unity-based connection: the participants felt connected beyond their local identities and felt a sense of “unity” (this feeling of “oneness,” and identity dissolution is vastly suggestive of ego-dissolutions); (2) Recognition and difference-based connection: a connection to and recognition of "the Other"; (3) Conflict-related revelations: there were reports of experiencing collective trauma and pain related to the conflict (Israel-Palestine) (some interviewees reported moments of strong empathy with the trauma of "the Other," this trigger of collective pain and trauma provides support for the existing claim of ayahuasca’s therapeutic potential for PTSD).

**Table 7:** Methods and administration of studies for ritualistic impacts

Study	# of doses	Method of administration	Length of study	Analysis used	# of people	Result
Roseman L et al. (2021) [50]	Variable	Interview	Variable	Grounded theory	31	Between 13 Arab Palestinians and 18 Jewish Israelis there were reports of: “1) Unity-Based Connection – collective events in which a feeling of unity and ‘oneness’ is experienced, whereby participants related to each other based upon a sense of shared humanity, and other social identities seemed to dissolve (such as national and religious identities). 2) Recognition and Difference-Based Connection – events where a strong connection was made to the other culture. These events occurred through the expression of the other culture or religion through music or prayers, which resulted in feelings of awe and reverence 3) Conflict-relatedrevelations – events where participants revisited personal or historical traumatic elements related to the conflict, usually through visions.”
Pontual AA et al. (2021) [51]	Variable	Interviews	Variable	Setting Questionnaire for the Ayahuasca Experience (SQAE)	19	The study provided significance for SQAE as a reliable tool to use for future measuring of setting in the role of ayahuasca
Pontual AA et al. (2022) [52]	Variable	Questionnaires	Variable	Setting Questionnaire for the Ayahuasca Experience (SQAE), The Mystical Experience Questionnaire (MEQ), and the Challenging Experience Questionnaire (CEQ)	2751	Ratings on SQAE negatively correlated with CEQ. Ratings on SQAE positively correlated with MEQ. The focus on the SQAE for both of the above results was social, comfort, infrastructure, and decoration
Apud I et al. (2023) [53]	Variable	Interviews	Variable	Zuckerman-Kuhlman-Aluja Personality Questionnaire	36	Questionnaire had significantly higher scores in impulsive sensation seeking, boredom susceptibility, and social warmth scales. Qualitative analysis showed emotional experiences (including social emotions such as love and empathy), corporal experiences, spiritual/transcendental experiences, personal experiences, and visions were most important to participants.
Uthaug MV et al. (2021) [54]	1	Interviews	1 day	None	30	Symptoms in both Ayahuasca drinkers and Placebo groups that did not drink ayahuasca but participated in the ceremony including the pre-diet prep, music, shaman guidance decreased.

However, this study brought in a new perspective where dealing with trauma is not only an inner-individual process. The study results emphasize that the ayahuasca group went beyond the interviewees’ identities, political opinions, and ongoing conflicts. There was a new form of social connection formed of which the biochemical aspect of ayahuasca was only part of it. In particular, the music was highlighted by most interviewees as the source through which they were able to open up and express themselves to the group, making the ceremony a space in which intercultural and interfaith exchange occurred, and this was intensified by ayahuasca. Another aspect was the vibration from the different languages and music that provoked visions [50]. A common experience described among ayahuasca users or aimed to be provoked during the ceremony is the “mystical experience.” This has been a growing interest for researchers in recent years and the importance it has for mental illnesses such as depression and anxiety. A cross-sectional study administered an online questionnaire to a total of 2,751 participants. These participants were classified based on the three available ceremony vehicles one can participate. The Setting Questionnaire for the Ayahuasca Experience (SQAE) was used to evaluate six dimensions of the setting characteristics. The SQAE was developed by Pontual et al. 2021 [51]. The six dimensions included were: social (how participants felt within the group), leadership (participants’ trust and feeling towards the organizer), decoration (how they felt in the space, infrastructure, comfort (how comfortable the space was), and instructions (how they were given preparatory instructions about the event). The Mystical Experience Questionnaire (MEQ) asked participants about the degree to which they had a certain experience like “oneness” and “unity.” The Challenging Experience Questionnaire (CEQ) included 26 items and seven factors: sadness, fear, death, insanity, isolation, physical suffering, and paranoia. The study found a negative correlation between SQAE and CEQ showing that comfortable, high-quality settings decreased challenges for participants. While there was a positive correlation between SQAE and ranking of mystical experiences. Specifically, music elements in a foreign language, religious music, and icaros had the highest positive correlation with mystical experience, similarly seen in the Palestine study. In some settings, participants are encouraged to participate, and even silence contributes to increasing closeness which is seen in both studies [52]. Table 7 summarizes some of these findings.

The music itself has been described to illuminate the participants down the road of emotional and mental states prompted by synesthesia. The medicine circle described by neo shaman was observed in a study including 36 participants at a Uruguayan psychotherapeutic center being treated for substance abuse distance. The ayahuasca group was compared to a control group with participants who had not participated in any psychedelic therapy. The results showed a statistically significant high score of social warmth. These results demonstrated that in addition to the biochemical effects of ayahuasca the community, music, and setting acted in synergy to elicit feelings of empathy, love, happiness, and unity [53].

The effect of the environment on the ayahuasca experience is evident and essential. To evaluate the extent to which the environment is important in the actual biochemical impact of ayahuasca a naturalistic observational study was performed with 30 participants: 16 assigned to placebo and 14 consumed ayahuasca. Assessments on depression, stress, and anxiety were performed pre- and post-ceremony. While the overall rating of all three was lower for both groups post-ceremony, the depression rating declined more in the placebo group. This is suggestive that non-pharmacological effects are indeed playing an essential role in the effect ayahuasca has on mental illnesses. However, it is important to note that the following study had participants who had previously taken ayahuasca and had some expectations going in, this could have contributed to their openness and consequently improvement in ratings. Moreover, both groups had similarly low ratings suggesting the dosing could not have been high enough [54].

An additional understanding of the importance of the ritual itself was demonstrated through multiple shamans interviewed on their understanding of the mechanism behind ayahuasca and the ceremony itself in treating eating disorders. A few shamans described patients with eating disorders feeling alienated, and disconnected from the community and self. The ritual itself allows them to feel connected/part of a group of individuals also wanting to “self-heal”. It allows participants to build on a foundation of well-being and seek a circle that will support them. Moreover, the concern of purging and dietary restriction which reinforces the negative compulsion in eating disorders was explained to be beneficial, a cleansing process when performed in the context of the ceremony [39]. Nonetheless, the importance of the setting and ritual itself is significant. Ayahuasca cannot be studied for its therapeutic potential without understanding this relationship. 

## Conclusions

This systematic review aimed to congregate the amount of recent relevant research available on the uses of ayahuasca for depression, anxiety, eating disorders, post-traumatic stress disorders, and overall well-being. The use of ayahuasca and the research supporting and detailing its effects suggests that there are multiple ways by which ayahuasca impacts the human body, mind, and spirit making it an impressive treatment option for the psychological disorders that plague the world today. The studies and hypotheses explored in this article suggest that ayahuasca is a powerful agent, and if used in the correct setting with the correct guidance, can prove as an alternative treatment for a variety of psychological disorders. The limitations to the use of ayahuasca as a standardized therapy are illustrated by the variety of questionnaires, illnesses, and methods of administration used in the recent research detailed in this paper. Concurrent strides in research on the use of ayahuasca have also been made, with more randomized placebo-controlled studies, and various cross-sectional studies being conducted in the past five years. This calls for increased control on the reporting and administration of ayahuasca with a longer time frame of following patients and possibly studying repeated doses to see the long-term effects of the therapy. As research for ayahuasca continues and is used as an experimental therapeutic drug the adaptation should not be a prescription pill but rather the traditional practice. It is vital to study, understand, and respect all aspects of the ritual, even the ones like emesis that do not traditionally fit in. It is not impossible to think that with increased research and more tightly regulated research conduction methods, ayahuasca could be a burgeoning holistic therapy for common psychological disorders. 

## References

[1] (2022). COVID-19 pandemic triggers 25% increase in prevalence of anxiety and depression worldwide. https://www.who.int/news/item/02-03-2022-covid-19-pandemic-triggers-25-increase-in-prevalence-of-anxiety-and-depression-worldwide.

[2] Group UNSD (2023). UN Sustainable Development Group. https://unsdg.un.org/.

[3] Walsh CA, Livne O, Shmulewitz D, Stohl M, Hasin DS (2022). Use of plant-based hallucinogens and dissociative agents: U.S. Time Trends, 2002-2019. Addict Behav Rep.

[4] Marijuana and hallucinogen use among young adults reached all time-high in 2021 (2023). Marijuana and hallucinogen use among young adults reached all time-high in 2021. young adults reached all.

[5] Batty D (2023). People ‘microdosing’ on psychedelics to improve wellbeing during pandemic. The Guardian.

[6] Berlowitz I, Egger K, Cumming P (2022). Monoamine oxidase inhibition by plant-derived β-carbolines; implications for the psychopharmacology of tobacco and ayahuasca. Front Pharmacol.

[7] Estrella-Parra EA, Almanza-Pérez JC, Alarcón-Aguilar FJ (2019). Ayahuasca: uses, phytochemical and biological activities. Nat Prod Bioprospect.

[8] van Oorsouw K, Toennes SW, Ramaekers JG (2022). Therapeutic effect of an ayahuasca analogue in clinically depressed patients: a longitudinal observational study. Psychopharmacology (Berl).

[9] (2023). Ayahuasca retreats & courses in Peru. https://www.ayahuascafoundation.org/.

[10] Organization WH: Depressive disorder (2023). Depressive disorder (depression). https://www.who.int/news-room/fact-sheets/detail/depression.

[11] Palhano-Fontes F, Barreto D, Onias H (2019). Rapid antidepressant effects of the psychedelic ayahuasca in treatment-resistant depression: a randomized placebo-controlled trial. Psychol Med.

[12] Fotiou E (2023). Shamanic tourism. ReVista: Harvard Review of Latin America.

[13] Zuloff-Shani AMD (2023). Celebrities are speaking out on psychedelics. Celebrities Are Speaking Out on Psychedelics. Psychology Today.

[14] Jaeger C (2023). ‘The unparalleled greatest feeling’ or risky drug? Inside the celebrity-loved psychedelic. https://www.smh.com.au/lifestyle/health-and-wellness/the-unparalleled-greatest-feeling-or-risky-drug-inside-the-celebrity-loved-psychedelic-20220331-p5a9oa.html.

[15] Haddaway NR, Page MJ, Pritchard CC, McGuinness LA (2022). PRISMA2020: An R package and shiny app for producing PRISMA 2020-compliant flow diagrams, with interactivity for optimised digital transparency and open synthesis. Campbell Syst Rev.

[16] Gonçalves J, Luís Â, Gradillas A (2020). Ayahuasca beverages: phytochemical analysis and biological properties. Antibiotics (Basel).

[17] Rossi GN, Guerra LT, Baker GB, Dursun SM, Saiz JC, Hallak JE, Dos Santos RG (2022). Molecular pathways of the therapeutic effects of Ayahuasca, a botanical psychedelic and potential rapid-acting antidepressant. Biomolecules.

[18] de Almeida RN, Galvão AC, da Silva FS (2019). Modulation of serum brain-derived neurotrophic factor by a single dose of ayahuasca: observation from a randomized controlled trial. Front Psychol.

[19] Inserra A (2018). Hypothesis: the psychedelic ayahuasca heals traumatic memories via a sigma 1 receptor-mediated epigenetic-mnemonic process. Front Pharmacol.

[20] Galvão AC, de Almeida RN, Silva EA (2018). Cortisol modulation by ayahuasca in patients with treatment resistant depression and healthy controls. Front Psychiatry.

[21] Galvão-Coelho NL, de Menezes Galvão AC, de Almeida RN (2020). Changes in inflammatory biomarkers are related to the antidepressant effects of Ayahuasca. J Psychopharmacol.

[22] Pallavicini C, Cavanna F, Zamberlan F (2021). Neural and subjective effects of inhaled N,N-dimethyltryptamine in natural settings. J Psychopharmacol.

[23] Timmermann C, Roseman L, Schartner M (2019). Neural correlates of the DMT experience assessed with multivariate EEG. Sci Rep.

[24] Alves CL, Cury RG, Roster K, Pineda AM, Rodrigues FA, Thielemann C, Ciba M (2022). Application of machine learning and complex network measures to an EEG dataset from ayahuasca experiments. PLoS One.

[25] Sarris J, Perkins D, Cribb L (2021). Ayahuasca use and reported effects on depression and anxiety symptoms: an international cross-sectional study of 11,912 consumers. J Affect Disord Rep.

[26] Zeifman RJ, Palhano-Fontes F, Hallak J, Arcoverde E, Maia-Oliveira JP, Araujo DB (2019). The impact of ayahuasca on suicidality: results from a randomized controlled trial. Front Pharmacol.

[27] Savoldi R Roazzi A, de Oliveira Sales RC (2023). Mystical and ego-dissolution experiences in ayahuasca and jurema holistic rituals: an exploratory study. Int J Psychol Relig.

[28] Dos Santos RG, Osório FL, Rocha JM (2021). Ayahuasca improves self-perception of speech performance in subjects with social anxiety disorder: a pilot, proof-of-concept, randomized, placebo-controlled trial. J Clin Psychopharmacol.

[29] Daldegan-Bueno D, Révész D, Morais PR, Barbosa PC, Maia LO (2022). Psychosocial and drug use assessment of regular vs. non-regular ayahuasca users in a brazilian sample: a web-based survey. Subst Use Misuse.

[30] van Oorsouw KI, Uthaug MV, Mason NL, Broers NJ, Ramaekers JG (2021). Sub-acute and long-term effects of ayahuasca on mental health and well-being in healthy ceremony attendants: a replication study. J Psychedelic Stud.

[31] Giovannetti C, Garcia Arce S, Rush B, Mendive F (2020). Pilot evaluation of a residential drug addiction treatment combining traditional amazonian medicine, ayahuasca and psychotherapy on depression and anxiety. J Psychoactive Drugs.

[32] Uthaug MV, van Oorsouw K, Kuypers KP (2018). Sub-acute and long-term effects of ayahuasca on affect and cognitive thinking style and their association with ego dissolution. Psychopharmacology (Berl).

[33] Rocha JM, Rossi GN, Osório FL, Hallak JE, Dos Santos RG (2022). Adverse effects after ayahuasca administration in the clinical setting. J Clin Psychopharmacol.

[34] dos Santos RG, Osório FL, Crippa JA, Hallak JE (2017). Anxiety, panic, and hopelessness during and after ritual ayahuasca intake in a woman with generalized anxiety disorder: a case report. J Psychedelic Stud.

[35] Barbosa PC, Tófoli LF, Bogenschutz MP (2018). Assessment of alcohol and tobacco use disorders among religious users of ayahuasca. Front Psychiatry.

[36] Daldegan-Bueno D, Maia LO, Massarentti CM, Tófoli LF (2022). Ayahuasca and tobacco smoking cessation: results from an online survey in Brazil. Psychopharmacology (Berl).

[37] Perkins D, Opaleye ES, Simonova H (2022). Associations between ayahuasca consumption in naturalistic settings and current alcohol and drug use: Results of a large international cross-sectional survey. Drug Alcohol Rev.

[38] Lawn W, Hallak JE, Crippa JA (2017). Well-being, problematic alcohol consumption and acute subjective drug effects in past-year ayahuasca users: a large, international, self-selecting online survey. Sci Rep.

[39] Williams M, Kingston Miller A, Loizaga-Velder A, Files N, Lafrance A (2023). "getting to the root": ayahuasca ceremony leaders' perspectives on eating disorders. J Psychoactive Drugs.

[40] Lafrance A, Loizaga-Velder A, Fletcher J, Renelli M, Files N, Tupper KW (2017). Nourishing the spirit: exploratory research on ayahuasca experiences along the continuum of recovery from eating disorders. J Psychoactive Drugs.

[41] Renelli M, Fletcher J, Tupper KW, Files N, Loizaga-Velder A, Lafrance A (2020). An exploratory study of experiences with conventional eating disorder treatment and ceremonial ayahuasca for the healing of eating disorders. Eat Weight Disord.

[42] Spriggs MJ, Kettner H, Carhart-Harris RL (2021). Positive effects of psychedelics on depression and wellbeing scores in individuals reporting an eating disorder. Eat Weight Disord.

[43] Ruffell SG, Netzband N, Tsang W (2021). Ceremonial ayahuasca in Amazonian retreats-mental health and epigenetic outcomes from a six-month naturalistic study. Front Psychiatry.

[44] Weiss B, Miller JD, Carter NT, Keith Campbell W (2021). Examining changes in personality following shamanic ceremonial use of ayahuasca. Sci Rep.

[45] Murphy-Beiner A, Soar K (2020). Ayahuasca's 'afterglow': improved mindfulness and cognitive flexibility in ayahuasca drinkers. Psychopharmacology (Berl).

[46] Pasquini L, Palhano-Fontes F, Araujo DB (2020). Subacute effects of the psychedelic ayahuasca on the salience and default mode networks. J Psychopharmacol.

[47] Kiraga MK, Mason NL, Uthaug MV, van Oorsouw KI, Toennes SW, Ramaekers JG, Kuypers KP (2021). Persisting effects of ayahuasca on empathy, creative thinking, decentering, personality, and well-being. Front Pharmacol.

[48] Soler J, Elices M, Dominguez-Clavé E (2018). Four weekly ayahuasca sessions lead to increases in "acceptance" capacities: a comparison study with a standard 8-week mindfulness training program. Front Pharmacol.

[49] MacKenzie MB, Abbott KA, Kocovski NL (2018). Mindfulness-based cognitive therapy in patients with depression: current perspectives. Neuropsychiatr Dis Treat.

[50] Roseman L, Ron Y, Saca A (2021). Relational processes in ayahuasca groups of Palestinians and Israelis. Front Pharmacol.

[51] Pontual AA, Tófoli LF, Collares CF, Ramaekers JG, Corradi-Webster CM (2021). The setting questionnaire for the ayahuasca experience: questionnaire development and internal structure. Front Psychol.

[52] Pontual AA, Tófoli LF, Corradi-Webster CM, van Oorsouw K, Delgado AR, Ramaekers JG (2022). The influence of ceremonial settings on mystical and challenging experiences occasioned by ayahuasca: a survey among ritualistic and religious ayahuasca users. Front Psychol.

[53] Apud I, Scuro J, Carrera I, Oliveri A (2023). Ayahuasca ritual, personality and sociality: observational research conducted in a substance use disorder rehabilitation center in Uruguay. J Psychoactive Drugs.

[54] Uthaug MV, Mason NL, Toennes SW (2021). A placebo-controlled study of the effects of ayahuasca, set and setting on mental health of participants in ayahuasca group retreats. Psychopharmacology (Berl).

